# Noninvasive early identification of durable clinical benefit from immune checkpoint inhibition: a prospective multicenter study (NCT04566432)

**DOI:** 10.1038/s41392-024-02060-3

**Published:** 2024-12-16

**Authors:** Xinghao Ai, Bo Jia, Zhiyi He, Junping Zhang, Minglei Zhuo, Jun Zhao, Zhe Wang, Jiexia Zhang, Zaiwen Fan, Xiaotong Zhang, Chong Li, Feng Jin, Ziming Li, Xia Ma, Hao Tang, Xiang Yan, Wei Li, Yuanyuan Xiong, Huan Yin, Rongrong Chen, Shun Lu

**Affiliations:** 1 Shanghai Lung Cancer Center, Shanghai Chest Hospital, Shanghai Jiao Tong University School of Medicine, Shanghai, China; 2 Key Laboratory of Carcinogenesis and Translational Research (Ministry of Education / Beijing), Department of Thoracic Medical Oncology, Peking University Cancer Hospital & Institute, Beijing, China; 3 Department of Pulmonary and Critical Care Medicine, The First Affiliated Hospital of Guangxi Medical University, Nanning, China; 4 Department of Thoracic Oncology, Cancer Center, Shanxi Bethune Hospital, Shanxi Academy of Medical Sciences, Tongji Shanxi Hospital, Third Hospital of Shanxi Medical University, Taiyuan, China; 5 Department of Thoracic Surgery, National Cancer Center / National Clinical Research Center for Cancer / Cancer Hospital & Shenzhen Hospital, Chinese Academy of Medical Sciences and Peking Union Medical College, Shenzhen, China; 6 State Key Laboratory of Respiratory Disease, National Clinical Research Center for Respiratory Disease, Guangzhou Institute of Respiratory Health, The First Affiliated Hospital of Guangzhou Medical University, Guangzhou, China; 7 Department of Pulmonary and Critical Care Medicine, Air Force Medical Center, PLA, Beijing, China; 8 Department of Pulmonary and Critical Care Medicine, Peking Union Medical College Hospital, Chinese Academy of Medical Sciences & Peking Union Medical College, Beijing, China; 9 Department of Pulmonary and Critical Care Medicine, Third Affiliated Hospital of Soochow University, First People’s Hospital of Changzhou, Changzhou, China; 10 Oncology Department, Qianjiang National Hospital, Chongqing, China; 11 Department of Respiratory and Critical Care Medicine, Beijing Youan Hospital, Capital Medical University, Beijing, China; 12 Department of Respiratory and Critical Care Medicine, Shanghai Changzheng Hospital, Naval Medical University, Shanghai, China; 13 Department of Thoracic Surgery, Peking University People’s hospital, Beijing, China; 14 Department of Respiratory and Critical Care Medicine, Second Affiliated Hospital of Xi’an Jiaotong University, Xi’an, China; 15 GenePlus-Beijing, Beijing, China

**Keywords:** Lung cancer, Tumour immunology, Tumour biomarkers

## Abstract

Immune checkpoint inhibitors (ICIs) have changed the treatment landscape for patients with non-small cell lung cancer (NSCLC). In spite of durable responses in some patients, many patients develop early disease progression during the ICI treatment. Thus, early identification of patients with no durable benefit would facilitate the clinical decision for these patients. In this prospective, multicenter study, 101 non-*EGFR/ALK* patients who received ICI treatment were enrolled after screening 328 stage III-IV NSCLC patients. At the date of cutoff, 83 patients were eligible for ICI efficacy evaluation, with 56 patients having progress-free survival (PFS) over 6 months, which was defined as durable clinical benefit (DCB). A multimodal model was established by integrating normalized bTMB, early dynamic of ctDNA and the first RECIST response. This model could robustly predict DCB with area under the curve (AUC) of 0.878, sensitivity of 79.2% at 86.4% specificity (accuracy = 80.0%). This model was further validated in the independent cohort of the DIREct-On study with AUC of 0.887, sensitivity of 94.7% at 85.3% specificity (accuracy = 90.3%). Patients with higher predict scores had substantially longer PFS than those with lower scores (training cohort: median PFS 13.6 vs 4.2 months, *P* < 0.001, HR = 0.24; validation cohort: median PFS 11.0 vs 2.2 months, *P* < 0.001, HR = 0.17). Taken together, these results demonstrate that integrating early changes of ctDNA, normalized bTMB, and the first RECIST response can provide accurate, noninvasive, and early prediction of durable benefits for NSCLC patients treated with ICIs. Further prospective studies are warranted to validate these findings and guide clinical decision-making for optimal immunotherapy in NSCLC patients.

## Introduction

Immune checkpoint inhibitors (ICIs) that block the inhibitory programmed death-ligand 1 (PD-L1) or programmed cell death 1 receptor (PD-1) have revolutionized cancer therapeutics, offering new hope to patients with various types of malignancies.^1–7^ Despite the significant potential of ICIs to improve patient survival, only a subset of patients would experience long-term benefit from this treatment, while other patients may develop early disease progression. This variability in response highlights the urgent need for robust, predictive biomarkers to stratify patients who are likely to benefit from ICI therapy.

However, currently used biomarkers, such as tumor mutation burden (TMB) or PD-L1 expression, have shown limited success in consistently predicting therapeutic response. It was estimated that the area under the curve (AUC) for predicting immunotherapy benefit using TMB or PD-L1 alone was typically around 0.6–0.7.^5,8^ Moreover, multivariable models based on molecular analyses of tumor biopsy tissue collected prior to treatment achieved modest improvements, with AUC ranging from 0.7 to 0.8,^9–14^ which was not sufficient for further clinical decision-making.

The complexity of the tumor microenvironment and intricate interactions with the immune system pose significant challenges in identifying a single biomarker that can robustly profile prognosis and prediction. It necessitates a more comprehensive and integrative approach to biomarker discovery and validation. With the advent of artificial intelligence (AI), AI-based methods have been widely employed to integrate multi-omics data to define meta-biomarkers. In this approach, genomics, radiomics, transcriptomics, epigenomics, pathomics, as well as real-world data were the most often used data to create more accurate predictive models. For instance, Ahn et al. developed a LightGBM algorithm based model using 19 clinical features at the beginning of treatment, and it showed better prediction performance compared to individual clinical feature and traditional multivariate logistic regression.^15^ Similarly, Vanguri et al. generated a multimodal model that incorporated features extracted from radiology, pathology, genomics and standard-of-care approved biomarkers. This model outperformed unimodal measures in predicting immunotherapy response with AUC of 0.80 in advanced non-small cell lung cancer (NSCLC).^16^ These studies underscore the potential of AI-driven multi-omics approaches in enhancing the accuracy of predictive models for ICI response.

In contrast to the static assessments of pre-therapeutic tissues samples, liquid biopsy with circulating tumor DNA (ctDNA) offered the advantage of assessing real-time molecular responses through rapid identification of primary resistance.^17^ Several studies have demonstrated the associations between reductions in ctDNA and prolonged survival in patients treated with ICIs for NSCLC.^18–20^ For example, Zhang et al. developed a “molecular response” matric using the ratio of on-treat variant allele frequency (VAF) to pretreatment VAF, derived from ctDNA assay. They found a strong association between “molecular response” and Response Evaluation Criteria in Solid Tumors (RECIST) response, with an AUC of 0.82.^21^ The BR.36 trial further validated the utility of ctDNA response in predicting RECIST response, demonstrating a sensitivity of 82%, specificity of 75% and a median lead time of 2.1 months.^22^ These findings highlight the potential of ctDNA as a dynamic and sensitive biomarker for monitoring treatment response and guiding clinical decisions.

In October 2020, Nabet et al. developed an excellent noninvasive multiparameter assay (DIREct-On) to predict patients who would achieve durable clinical benefit (DCB, over 6 months). Pre-treatment ctDNA, peripheral CD8 T cell levels, and early ctDNA dynamics were integrated in the DIREct-On model. This integrated approach achieved high accuracy (accuracy = 92%, AUC = 0.93) for predicting DCB in multiple retrospective cohorts.^23^ Notably, the DIREct-On model outperformed any individual feature or models based solely on pretreatment features. Inspired by the DIREct-On study, we designed this multicenter prospective observational clinical trial to explore multimodal models that were more feasible in the clinical routine for predicting DCB of ICI treatments in non-*EGFR/ALK* NSCLC patients. In this study, we would address several key questions: 1. Could pretreatment CD8 T cell levels be replaced by other parameters, given that it was the least contributing factor in the DIREct-On model and it required additional RNA sequencing of immune cells? 2. The feasibility of combining ctDNA with early radiographic response and how effective it might be, considering that the recently published BR.36 prospective study showed that ctDNA response at the third cycle of pembrolizumab could predict later RECIST response with a sensitivity of 82% and a specificity of 75%.^22^ 3. Which matric change in VAF or ctDNA concentration - could better predict the ICI response? By addressing these questions, we aim to develop a more practical and accurate multimodal model that can be readily implemented in clinical practice to optimize the selection and monitoring of ICI therapy in non-*EGFR/ALK* NSCLC patients. Specifically, we integrated ctDNA profiling data (including bTMB, VAF or ctDNA concentration) from pre- and early on-treatment blood samples, circulating immune cells derived from pre-treatment blood samples, and early radiographic response to develop our model. We also use the published DIREct-On study as an independent external validation cohort to validate our model. This approach holds the promise of improving patient outcomes and reducing unnecessary treatment-related costs and side effects.

## Results

### Patient characteristics

**Fig. 1:**
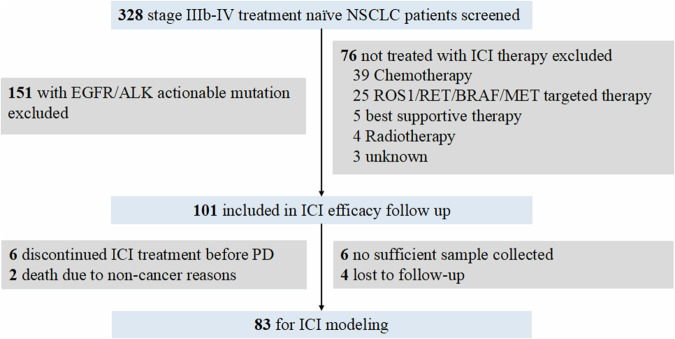
Overview of the study design. A total of 328 NSCLC patients were screened and 101 patients were enrolled in this study. However, 6 patients discontinued ICI treatment before PD mainly due to side effects, 2 patients died due to non-cancer reasons, 6 patients failed to collect sufficient blood samples at preset time and 4 patients were lost to follow-up. Finally, 83 NSCLC patients were included for further analysis

In the multicenter prospective observational study, 328 advanced NSCLC patients from 14 medical centers were screened to exclude *EGFR*/*ALK* mutant patients. There were 132 patients with *EGFR* actionable mutation and 19 patients with *ALK* fusion identified. For the 177 patients without actionable *EGFR*/*ALK* mutation, chemotherapy, ROS1/RET/BRAF/MET targeted therapy, best supportive therapy, and radiotherapy were performed in 39, 25, 5, and 4 patients respectively. With 3 patients withdrawing from the study, finally, we had 101 patients who underwent 1st line immunotherapy included in the further study (Fig. 1).

Of the 101 patients, 6 patients discontinued ICI treatment before PD mainly due to side effects, 2 patients died due to non-cancer reasons, so they were excluded from further analysis. Moreover, 6 patients failed to collect sufficient blood samples at preset time and 4 patients lost to follow-up were excluded. Finally, 83 NSCLC patients were included for subsequent analysis. The median age at diagnosis was 65.0 years old (range, 40.0–78.0) with 72 male patients (86.7%), and 69 at stage IV (83.1%). Fifty patients were adenocarcinoma, followed by 27 squamous-cell carcinoma, 4 low/poor differentiated carcinoma, and 1 adenosquamous carcinoma. For the 1st line ICI regimen, 66 patients received ICI combined with chemotherapy, 12 patients received ICI monotherapy, and 5 patients were combined with anti-angiogenesis therapy (Table 1).

**Table 1:** Patient characteristics (*N* = 83)

	*n* = 83
Age, years	
Mean (SD)	64.6 (8.0)
Median (min-max)	65 (40–78)
Gender, *n* (%)	
Male	72 (86.7)
Female	11 (13.3)
Smoking, *n* (%)	
No	19 (22.9)
Yes	62 (74.7)
Unknown	2 (2.4)
Histology, *n* (%)	
Adenocarcinoma	50 (60.3)
Squamous-cell carcinoma	28 (33.7)
Others	5 (6.0)
Tumor stage, *n* (%)	
III	14 (16.9)
IV	69 (83.1)
Baseline metastasis site, *n* (%)	
Bone	29 (34.9)
Lung	25 (30.1)
Brain	11 (13.3)
Pleura	8 (9.6)
Adrenal	5 (6.0)
Liver	2 (2.4)
Others	12 (14.5)
ICI therapy regimen, *n* (%)	
ICI+chemo	66 (79.5)
Monotherapy	12 (14.5)
ICI+antiangiogenesis	4 (4.8)
ICI+chemo+antiangiogenesis	1 (1.2)
Progression, *n* (%)	
Yes	49 (59.0)
No	34 (41.0)
Progression site, *n* (%)	
Lung	27 (32.5)
Brain	6 (7.2)
Lymph node	7 (8.4)
Bone	3 (3.6)
Liver	2 (2.4)
Others	4 (4.8)
Follow-up time, months, *n* (%)	
Not progressed, median (min-max)	11.1 (6.0–30.9)
Progressed, median (min-max)	4.5 (0.7–23.3)
Clinical outcome, *n* (%)	
DCB	56 (67.5)
NDB	27 (32.5)

At the date of December 15th 2023, 49 patients (59.0%) progressed and all the other 34 patients were followed over 6 months, with the median follow-up of 11.1 months (6.0–30.9 months). The disease progression happened in the lung of 26 patients, lymph node of 8 patients, brain of 6 patients, bone of 3 patients, liver of 2 patients, and other sites of 4 patients. Taken together, 56 patients (67.5%) had a progression-free survival (PFS) of at least 6 months and achieved DCB, and 27 patients progressed in 6 months and defined as no durable benefit (NDB) (Table 1).

### Pre-treatment features associated with ICI response

**Fig. 2:**
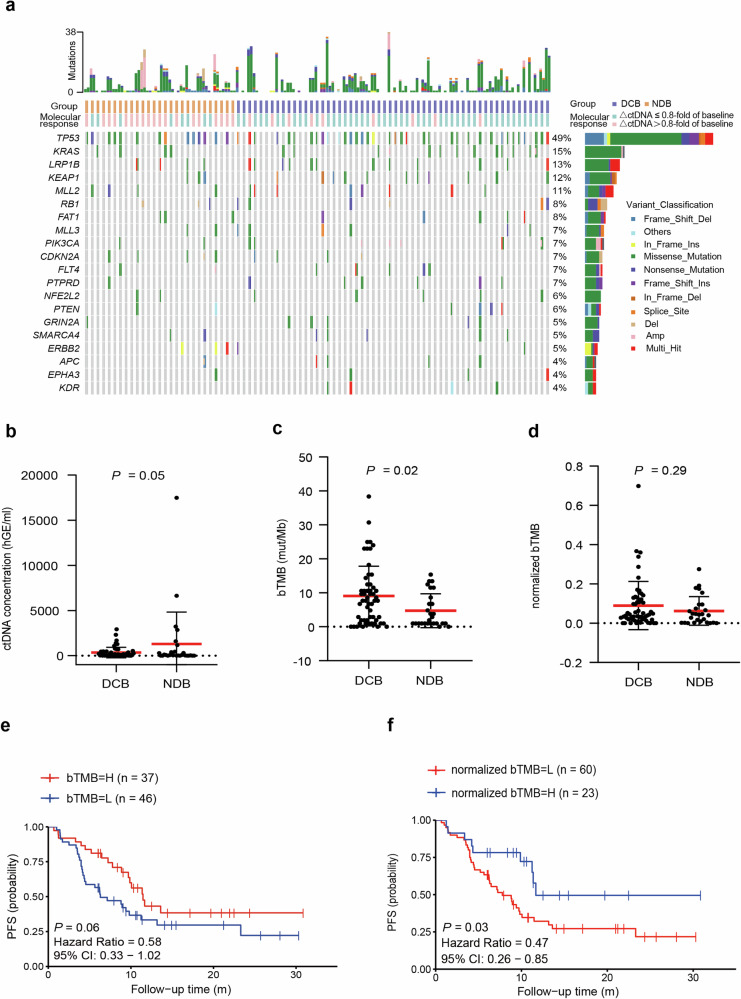
Pre-treatment features associated with DCB. **a** The top 20 genes detected by liquid biopsy pre- and early on-treatment were shown for the 83 patients. The prevalence of alterations in each gene is listed on the right. Mutation counts for each sample are shown at the top, followed by rows indicating the benefit of treatment and ctDNA molecular response. Patients who achieved DCB had lower ctDNA concentration (**b**) but higher bTMB level (**c**) than those who achieved NDB. **d** Patients who achieved DCB had higher normalized bTMB level compared to those achieved NDB. **e** Patients with high bTMB had longer PFS than those with low bTMB (median 11.5 months versus 6.3 months). **f** Patients with high normalized bTMB had longer PFS compared to patients with low normalized bTMB (median 11.7 months versus 8.8 months). PFS: progression-free survival; HR hazard ratios, CI confidence intervals, bTMB blood-based TMB, DCB durable clinical benefit, NDB no durable benefit. The red horizontal bar in the (**b**–**d**) represents the mean and error bar represent ± SD; *P* < 0.05 represents statistical significance

Pre-treatment ctDNA was detected in 94.0% of patients (78/83) with *TP53*, *KRAS*, *LRP1B*, *KEAP1*, and *MLL2* as the top 5 mutated genes (Fig. 2a). There were no enriched mutations in either DCB or NDB group. As expected, we found numerically lower ctDNA concentration and significantly higher bTMB in the DCB cohort (Fig. 2b, c).^24–26^ When using the ratio to integrate the ctDNA concentration and bTMB as ctDNA-normalized bTMB (normalized bTMB), we found no difference between DCB and NDB patients (Fig. 2d). While bTMB was not associated with PFS even with the best cutoff at 6.720 (Fig. 2e), patients with higher normalized bTMB (cutoff at 0.0975) had significantly better ultimate clinical outcomes (median PFS: 11.7 vs 8.8 months, *P* = 0.03, hazard ratio [HR]: 0.47, 95% CI: 0.26–0.85, Fig. 2f).

Inspired by the DIREct-On model,^23^ we also profiled peripheral blood mononuclear cell (PBMC) transcriptomes (*n* = 78). Unexpectedly, we observed no significant difference between the DCB and NDB groups, including circulating CD8 T and other immune cells (supplementary Fig. 1a, b). Patients with higher activated NK cells had borderline longer PFS than those with lower activated NK cells (*P* = 0.0496, HR: 0.57, 95% CI: 0.31–1.04, supplementary Fig. 1c). And activated NK cells alone had limited classification accuracy for predicting durable benefit (AUC = 0.633, supplementary Fig. 1d).

Separately, neither PD-L1 (*n* = 71) nor tTMB (*n* = 28) was related to the PFS of immunotherapy^3,27,28^ (supplementary Fig. 2a–d). Overall, these data suggest that pretreatment normalized bTMB and circulating activated NK cells can serve as potential predictors of ICI response.

### Early on-treatment ctDNA dynamics are associated with ICI response

**Fig. 3:**
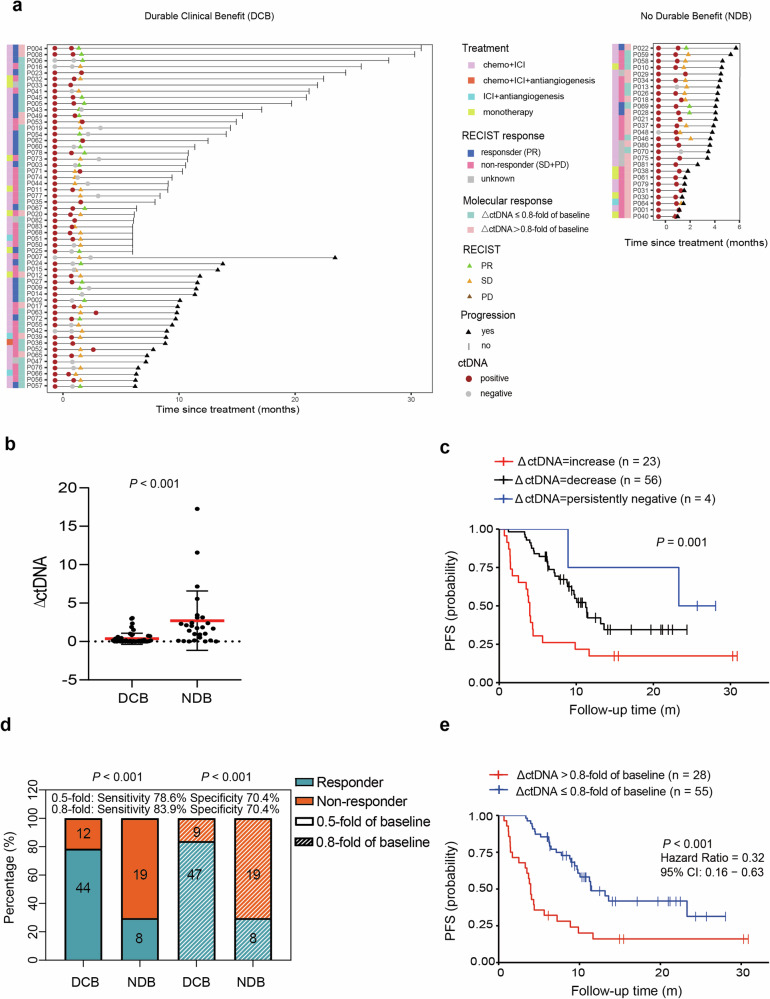
Early on-treatment ctDNA dynamics predicts response to ICI. **a** Swimmer plots display the timing of RECIST response assessment, molecular response and PFS for each patient. **b** Compared with DCB cohort, the ΔctDNA was significantly lower in the DCB group. The red horizontal bar represents the mean and error bar represent ±SD. **c** Patients with increased ctDNA had shorter PFS than those with decreased and persistently negative ctDNA (median 4.0 months versus 11.2 months versus 25.7 months). **d** The ΔctDNA had good sensitivity and specificity to identify patients with DCB. **e** Patients who achived a molecular response had longer PFS than those of non-molecular responders (median 11.5 months versus 4.0 months). DCB durable clinical benefit, Chemo chemotherapy, ICI immune checkpoint inhibitor, PR partial response, SD stable disease, PD disease progression, PFS progression-free survival. *P* < 0.05 represents statistical significance

In our cohort, most patients (89.1%, 74/83) had the 2nd blood samples collected after 1 or 2 cycles of ICI treatment, with a median of 26 days from the initial of ICI treatment (14–97 days) (Fig. 3a). We surveyed the association between DCB and ctDNA dynamics (ΔctDNA) as previously reported.^19,23,29,30^ The ΔctDNA was significantly lower in the DCB group (Fig. 3b). With ΔctDNA = 1 as the cut-point, ctDNA was decreased in 50 (89.3%, 50/56) patients of DCB group, and increased in 17 (63.0%, 17/27) of NDB patients. Moreover, patients with ctDNA persistently undetected or decreased had significantly longer PFS (median PFS: 25.7 vs 11.2 vs 4.0 months, *P* = 0.001, Fig. 3c). Considering the intra-day ctDNA variation, we then used the same cut-point of 50% decrease in ctDNA concentration as in the DIREct-On study,^23^ and found ctDNA burden dropped in 44 (78.6%, 44/56) of patients achieving DCB while it did not meet the 0.5-fold threshold in 19 (70.4%, 19/27) of NDB patients (*P* < 0.001; Fig. 3d). We then defined a threshold of 80% decrease in ctDNA concentration from pre-treatment as the “ctDNA molecular response”. Thus, 47 (83.9%,47/56) of patients achieving DCB were defined as ctDNA molecular response, and 19 (70.4%, 19/27) of NDB patients were defined as ctDNA non-responder (*P* < 0.001; Fig. 3d). Furthermore, early ctDNA dynamics outperformed all individual pre-treatment factors (supplementary Fig. 1d; AUC = 0.824) and patients with ctDNA molecular responses had significantly better clinical outcomes (median PFS: 11.5 months vs 4.0 months, HR: 0.32, 95% CI: 1.27–5.40, *P* < 0.001, Fig. 3e). Thus, early ctDNA molecular response was a promising approach for early response assessment. However, given that the accuracy rate was less than 75%, further improvements in predicting ultimate clinical outcomes would be desirable.

As part of the study’s exploratory analyses, we evaluated the predictive value of VAF change (ΔVAF) for DCB. Indeed, patients with low ΔVAF showed better PFS than those with high ΔVAF (supplementary Fig. 3a). We also defined a threshold of 80% decrease in VAF from pre-treatment as a “VAF response”. The sensitivity (83.9%) produced by VAF response was equal to that of ΔctDNA, but the specificity (55.6%) was lower than that of ΔctDNA (supplementary Fig. 3b). Taken together, our results indicate that ΔctDNA is more robust than ΔVAF in predicting benefit from immunotherapy.

### Early imaging RECIST response can predict DCB with high specificity but low sensitivity

**Fig. 4:**
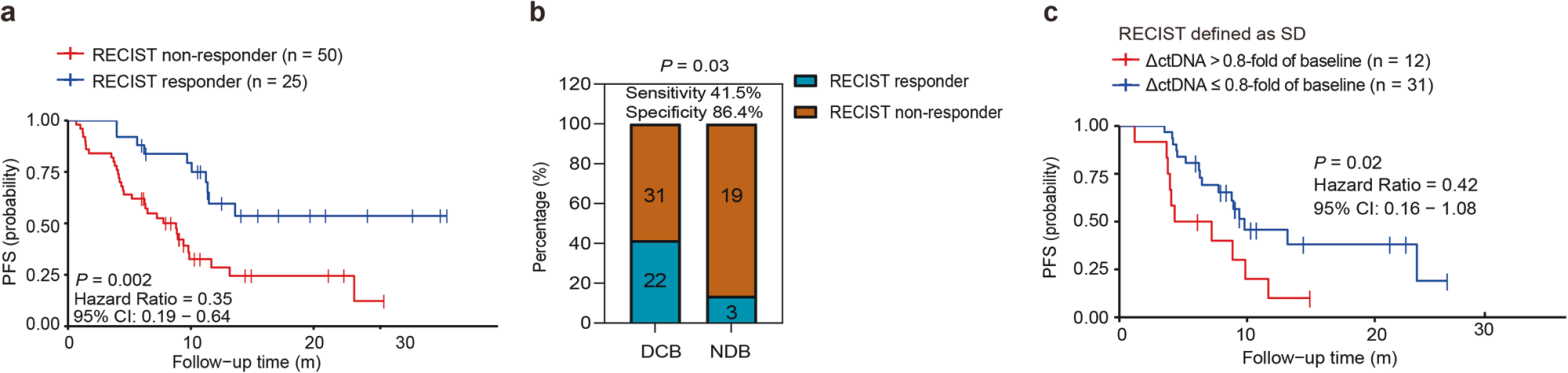
Early imaging RECIST evaluation predicts response to ICI. **a** Patients with RECIST responder (PR) had longer PFS than those with RECIST non-responder (SD + PD, median unreached versus 8.8 months). **b** Early RECIST response had good specificity, but low sensitivity to predict DCB. **c** Patients classified as molecular responders had longer PFS than nonmolecular responders in the cohort which RECIST were defined as SD (median 9.8 months versus 5.8 months). RECIST The response evaluation criteria in solid tumors, PR partial response, SD stable disease, PD disease progression, PFS progression-free survival, DCB durable clinical benefit. *P* < 0.05 represents statistical significance

A total of 75 patients had early imaging RECIST response assessment available (within 2 months of the ICI treatment, mainly after 2 cycles of ICI treatments), of which 25, 43, and 7 patients had a partial response (PR), stable disease (SD), and progression disease (PD) respectively. Patients who achieved RECIST response (PR, *n* = 25) had longer PFS than that of non-responders (SD/PD, *n* = 50; median PFS: undefined vs 8.8 months, HR: 0.35, 95% CI: 0.19–0.64, *P* = 0.002, Fig. 4a). However, the sensitivity of the early imaging RECIST response for predicting DCB was 41.5% (95% CI: 28–56%), the specificity was 86.4% (95% CI: 65–97%), and the prediction accuracy was 54.7% (Fig. 4b).

We further analyzed whether ctDNA molecular response could stratify the 43 SD patients. As expected, patients classified as ctDNA molecular responders had longer PFS than non-responders (median PFS: 9.8 vs 5.8 months, HR: 0.42, 95% CI: 0.16–1.08, *P* = 0.02, Fig. 4c).^31^

### Multimodal model for predicting response to ICIs

**Fig. 5:**
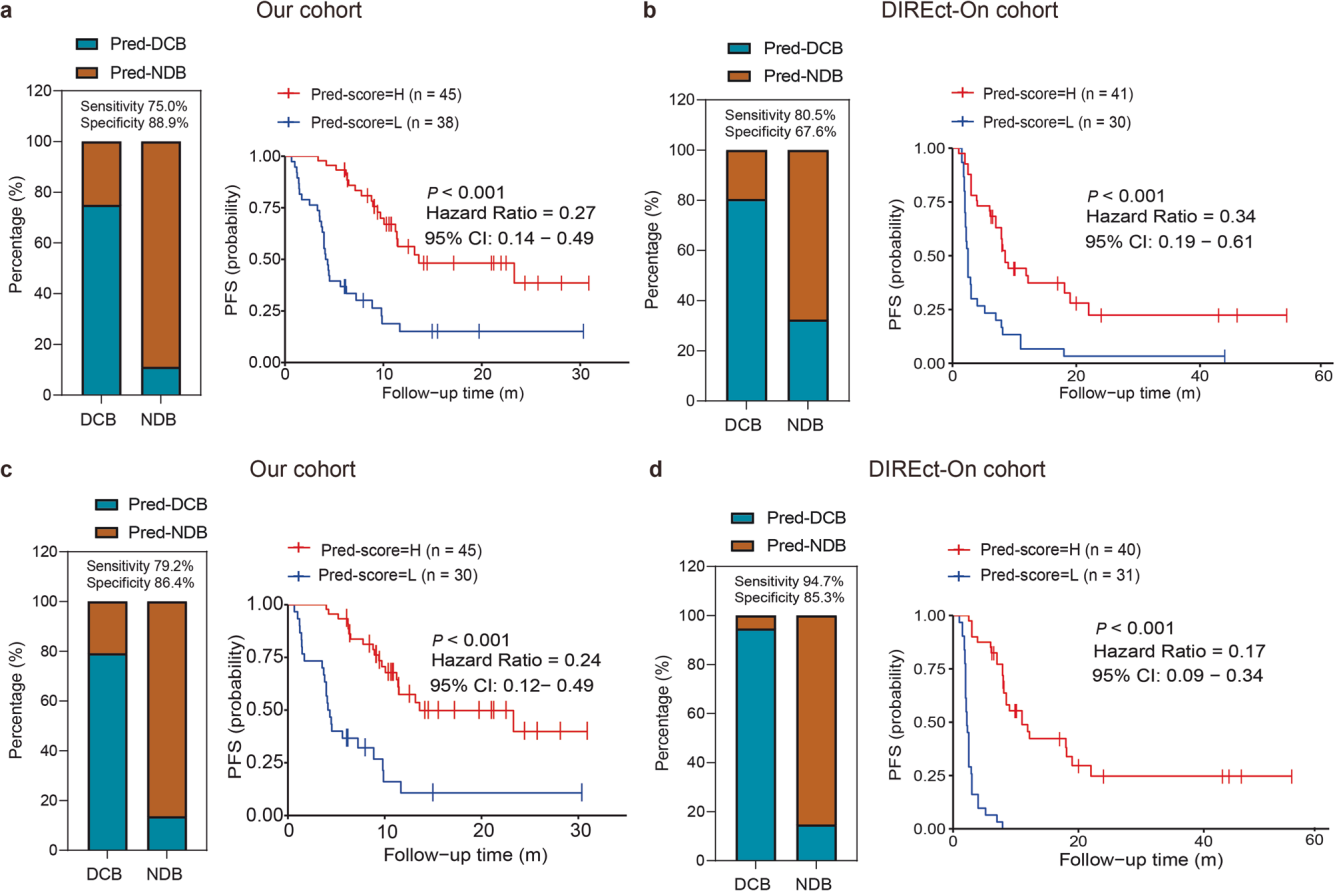
Multimodal model enables fully noninvasive outcome classification. **a** Two parameters (normalized bTMB and ΔctDNA) were used to build a model for predicting DCB in our discovery cohort, with a sensitivity of 75.0% and a specificity of 88.9%. Stacked column chart showed the proportion of patients predicted to achieve DCB (Pred-DCB) or NDB (Pred-NDB) by the model. Patients with higher predict scores had significantly longer PFS than those with lower scores (median 13.6 months versus 4.3 months). **b** Performance of the two parameters model in DIREct-On validation cohort, achieving a sensitivity of 80.5% and a specificity of 67.6%. Stacked column chart showing the proportion of patients with Pred-DCB or Pred-NDB. Patients with higher predict scores had longer PFS than those with lower scores (median 8.5 months versus 2.5 months). **c** Three parameters (normalized bTMB, ΔctDNA and the first RECIST response) were used to build a model for predicting DCB in our discovery cohort, with a sensitivity of 79.2% and a specificity of 86.4%. Stacked column chart showing the proportion of patients with Pred-DCB or Pred-NDB. Patients with higher predict scores had significantly longer PFS than those with lower scores (median 13.5 months versus 4.2 months). **d** Performance of the three parameters model in DIREct-On validation cohort, achieving a sensitivity of 94.7% and a specificity of 85.3%. Stacked column chart showing the proportion of patients with Pred-DCB or Pred-NDB. Patients with higher predict scores had longer PFS than those with lower scores (median undefined versus 2.2 months). bTMB blood-based TMB, DCB durable clinical benefit, NDB no durable benefit, RECIST The response evaluation criteria in solid tumors, PFS progression-free survival; *P* < 0.05 represents statistical significance

We then combined normalized bTMB with early on-treatment ctDNA dynamic in the context of a multiparameter predictor of ICI benefit since they were independent (supplementary Fig. 4). As expected, ΔctDNA emerged as the strongest prognostic factor for the outcome (supplementary Table 1). Eighty-three patients from our cohort (training cohort) and 75 patients from DIREct-On study (validation cohort)^23^ were used to construct and validate a logistic regression model. As a result, this model was able to predict DCB with AUC of 0.854 (supplementary Fig. 5a), and achieved 75.0% sensitivity (classifying DCB) and 88.9% specificity (accuracy = 79.5%, Fig. 5a). Moreover, this model had similar performance in the validation cohort, with AUC of 0.798 (supplementary Fig. 5b), sensitivity of 80.5% and specificity of 67.6% (accuracy = 74.7%, Fig. 5b). And the model had significantly better predictive performance than each individual metric (supplementary Fig. 5c, d). Patients with higher predict scores had substantially longer PFS than those with lower scores (training cohort: median PFS 13.6 vs 4.3 months, *P* < 0.001, HR = 0.27, Fig. 5a; validation cohort: median PFS 8.5 vs 2.5 months, *P* < 0.001, HR = 0.34, Fig. 5b).

Furthermore, adding RECIST response improved the performance of the model, with AUC improving to 0.878 (supplementary Fig. 5e), sensitivity to 79.2% and specificity to 86.4% (accuracy = 80.0%, Fig. 5c) in the training set. Meanwhile, the AUC of the external validation set improved to 0.887 (supplementary Fig. 5f), with sensitivity of 94.7% and specificity of 85.3% (accuracy = 90.3%, Fig. 5d). As expected, patients with higher predict scores had substantially longer PFS than those with lower scores (training cohort: median PFS 13.6 vs 4.2 months, *P* < 0.001, HR = 0.24, Fig. 5b; validation cohort: median PFS 11 vs 2.2 months, *P* < 0.001, HR = 0.17, Fig. 5d). Interestingly, adding RECIST response increased the sensitivity from 75.0% to 79.2% with a trade-off in specificity from 88.9% to 86.4% in our training cohort. However, both sensitivity (from 80.5% to 94.7%) and specificity (from 67.6% to 85.3%) were improved in the validation cohort. We speculated that it was because ΔctDNA was the major contributor to the model, followed by RECIST response and normalized bTMB (supplementary Table 1).

In addition, we also explored whether adding activated NK cells to the model could further improve the performance. However, adding activated NK cells impaired the predictive ability of the model (supplementary Fig. 6a, b), which was probably due to the previous observation that activated NK cells alone had limited classification accuracy for predicting durable benefit (AUC = 0.633, supplementary Fig. 1d).

Overall, these results suggested that our logistic regression model integrated RECIST response, normalized bTMB and ctDNA kinetics during treatment could accurately predict which patients will achieve DCB from ICI.

## Discussion

There is an unmet clinical need to evaluate therapeutic response early in the course of immunotherapy and guide further decision-making. However, currently used biomarkers such as PD-L1, TMB, or multivariable models based on molecular analyses of tumor tissue biopsy collected prior to treatment were not sufficiently accurate to identify all potential responders to PD-(L)1 blockade-based ICI in NSCLC.^5,8–14^ In this prospective multicenter study (NCT04566432), we comprehensively analyzed parameters associated with DCBs from ICI in advanced wild-type EGFR/ALK NSCLC patients. We developed a noninvasive response classifier that incorporates early on-treatment ctDNA response, normalized bTMB and early imaging data, which could classify patients with durable benefit from immunotherapy with high accuracy and validated it in an independent cohort from the DIREct-On study.^23^

In the DIREct-On model, pretreatment CD8 T cell levels could classify DCB or NDB patients with an accuracy of 70% and the removal of CD8 T cell fractions could decrease the performance (NRI = −1.13, *P* < 0.001).^23^ However, in our study, circulating CD8 T and other immune cells did not differ significantly between patients achieving or failing to achieve DCB from ICI, though we also profiled leukocyte transcriptomes in patients with available preserved PBMC specimens using RNA sequencing and applied CIBERSORTx^32,33^ to quantify the relative proportions of major leukocyte subpopulations. On the contrary, we found patients with lower baseline circulating activated NK cell levels tended to have longer PFS from ICI, this was somehow consistent with observations in melanoma responders to immunotherapy,^34^ and fewer activated NK cells in circulation might reflect greater gathering of activated NK cells to tumor deposits in patients with immunogenic tumors, just as circulating CD8 T cells in the DIREct-On model.^23^ However, activated NK cells alone had limited classification accuracy for predicting durable benefit (AUC = 0.633, supplementary Fig. 1d) and adding activated NK cells impaired the model’s performance (supplementary Fig. 6a, b). The discrepancy of the two studies might be due to several reasons. Firstly, most patients in the DIREct-On model (60/75) were treated with ICI without chemotherapy,^23^ while most patients were treated with ICI plus chemotherapy (67/83) in our cohort. Secondly, our study was mostly carried out during the COVID-19 pandemic, we could not exclude the potential effect of the virus infection, as both NK cells and CD8 T cells would respond to the virus, especially there was only borderline difference in activated NK or CD8 T cells between the DCB and NDB groups. Considering the time and effort requirements for separating PBMC and RNA sequencing, as well as the variable and complex response of immune cells to the environment, models including leukocyte transcriptome data might be complicated and not so stable.

Several publications have demonstrated that ctDNA changes of 1–4 cycles after therapy initiation can classify response to ICI in NSCLC with modest accuracy (AUC = 0.65–0.75).^18,19,29^ In our exploratory study, we compared the performance of ΔctDNA and ΔVAF in predicting benefits from immunotherapy. As patients with higher tumor burden are known to respond less well to ICIs,^35,36^ ΔctDNA concentration could better reflect the whole tumor burden than ΔVAF as indicated in our study. Moreover, most patients (74/83) in our study had the 2nd plasma sample collected at 1–2 cycles, which is a little later than patients from DIREct-On model, probably, this might shift the best ΔctDNA cutoff from 50 to 80%. Besides the DIREct-On study, other research efforts have utilized genetics, digital pathology, radiomics features, and artificial intelligence to develop multimodal prediction models for immunotherapy response.^15,37–40^ While the AUC for predicting DCB ranged from 0.75 to 0.91, almost all of these studies relied on retrospective data and lacked prospective validation cohorts. Furthermore, models based solely on pretreatment baseline features generally demonstrated inferior performance compared to those that integrated on-treatment features, a finding consistent with our study.

The high specificity of the first imaging evaluation indeed improved the performance of our model. We acknowledged that radiologic surveillance of NSCLC patients treated with PD-(L)1 blockade was useful, especially for those patients with early PR or PD, as 22 of the 25 PR patients (88%) achieved DCB and all the 7 PD patients were NDB. We found that among patients achieving SD at the first scan, about one-third (27.9%; 12/43) did not ultimately achieve DCB similar to previous reports.^23,41^ ΔctDNA could separate these SD patients as well. This finding could help to discriminate pseudo-progression population and SD patients who can not ultimately achieve DCB thus early treatment adjustment might be helpful. We speculated that our findings could have important value in the neoadjuvant therapy setting as well to help in early time to predict clinical benefit and probability of radical resection.

Limitations of our study included the use of a heterogeneous PD-(L)1 regimen among patients in this prospective study (Table 1), with 79.5% receiving combined ICI and chemotherapy, 14.5% ICI monotherapy, 4.8% combined ICI and antiangiogenesis therapy, and 1.2% combined ICI, chemotherapy and antiangiogenesis therapy. Despite this heterogeneity, our model performed consistently well across different PD-(L)1 regimens. Secondarily, 6 patients were excluded due to missed sample collection at required timepoint and 4 patients lost to follow-up during the COVID-19 pandemic (Fig. 1). Thus, the final cohort had only 83 patients included even though we had screened 328 patients. Finally, though our model was validated by the DIREct-On model cohort (*n* = 72),^23^ further validation in larger real-world cohorts would still be necessary in real-world clinical settings.

In summary, our study generated an accurate classifier that could predict the long-term benefit of ICIs. This model could help address the unmet need for improving personalized therapy for patients treated with ICIs in both systemic and neoadjuvant settings by incorporating data from baseline, early on-treatment ctDNA testing, and imaging evaluation results. Further prospective interventional studies are warranted to validate the model in guiding clinical decision-making for optimal immunotherapy NSCLC patients.

## Materials and methods

### Patient recruitment and sample collection

A total of 328 Chinese patients with stage III or IV NSCLC from 14 hospitals were prospectively screened with next generation sequencing (NGS) or PCR based tests during January 2021 to June 2023. After excluding 151 patients with *EGFR*/*ALK* mutant and 76 patients who did not receive 1st line ICI treatment, 101 patients were included in the study. Twenty-milliliter peripheral blood samples were collected in Streck tubes from each patient before the first treatment, and early on-treatment timepoint (1–3 cycles of ICI treatments). After the sample was collected and labeled, it was immediately transported to the central laboratory (Geneplus-Beijing, Beijing), with a maximum allowable transportation time of 3 days. Upon arrival of the blood sample, plasma and PBMCs were isolated for further experiments. Radiographic evaluation was performed regularly upon physicians’ request. Response was quantified using RECIST v1.1,^42^ patients with complete response (CR) or partial response (PR) at the first imaging evaluation were defined as RECIST responders while others were non-responders. PFS was defined as the time elapsed between therapy initiation and disease progression or death. DCB was defined as progression-free for at least 6 months since immunotherapy; NDB was defined as progression or death within 6 months. All patients provided written informed consent to participate in the study. This study was performed under a protocol approved by the Institutional Review Board of Shanghai Chest Hospital (KS2017). All the procedures conformed to the principles of the Helsinki Declaration (Supplementary File 1). The trial has been registered with the number NCT04566432.

### RNA extraction and sequencing

PBMCs were isolated by Ficoll-gradient centrifugation (Ficoll-Paque Plus, GE Healthcare) whenever possible, and samples were stored at −80 °C immediately after collection until further analysis. The RNA isolation of tissue samples was performed using Rneasy Mini Kit (Qiagen). Constructing sequencing library, sequencing, and quality control for FASTQ data were performed following the protocol by Owens et al.,^43^ then clean data were matched to the human genome (GRCh37) by operating STAR alignment tool (V.2.7.6a)^44^ as previously reported.^45^

### DNA extraction and library preparation

Tumor DNA was extracted from formalin-fixed, paraffin-embedded (FFPE) tumor tissue specimens using the ReliaPrepTM FFPE gDNA Miniprep System (Promega, Madison, WI, USA). Cell-free DNA (cfDNA) was isolated using the QIAamp Circulating Nucleic Acid Kit (Qiagen, Hilden, Germany). The germline genomic DNA from white blood cells was isolated using the QIAamp DNA Blood Mini Kit (Qiagen, Valencia, CA, USA). The DNA concentration was measured using a Qubit fluorometer and the Qubit dsDNA HS (High Sensitivity) Assay Kit (Invitrogen, Carlsbad, CA, USA). The size distribution of the cfDNA was assessed using an Agilent 2100 BioAnalyzer and a DNA HS kit (Agilent Technologies, Santa Clara, CA, USA) as previously reported.^46^

Sequencing libraries were prepared using the KAPA Library Preparation Kit (Kapa Biosystems, Wilmington, MA, USA). A custom-designed panel covering ~1.5 Mbp genome and targeting 1021 cancer-related genes was used for hybridization enrichment with DNA libraries, and then sequenced using Gene+ Seq 2000 instrument (GenePlus-Beijing).^45,46^

### Targeted next-generation sequencing and genomic data analysis

After removing adapters and low-quality reads, the clean reads were mapped to the human reference genome (hg19) using BWA18 (version 0.7.12-r1039). The Picard software MarkDuplicates (v4.0.4.0; Broad Institute, Cambridge, MA, USA) was used for realignment and recalibration. Somatic single nucleotide variants were determined by MuTect and NChot (Geneplus-Beijing, inhouse). Small insertions and deletions (indels) were called using GATK (v3.6-0-g89b7209; Broad Institute). CONTRA (2.0.8) was employed to detect copy number alterations. A self-developed algorithm NCsv (v0.2.3 Geneplus-Beijing, inhouse) was used to identify structural variations (SVs). All final candidate variants were manually verified with the integrative genomics viewer browser. Somatic alterations were filtered with matched patient’s whole blood controls to remove germline mutations.^46^

Then, tumor mutational burden (tTMB) and blood-based TMB (bTMB) were calculated as the number of all nonsynonymous mutations per megabase (Muts/Mb) of genome examined as described previously.^47,48^ Tumor PD-L1 expression was assessed using the PD-L1 IHC 22C3 pharmDx assay (Dako) upon request.

### ctDNA molecular response evaluation

The ctDNA concentration was expressed in haploid genome equivalents (hGE) per mL of plasma (hGE/mL) and was calculated by multiplying the maximal VAF of detected mutations by the plasma cfDNA concentration and dividing by 3.3, with the assumption that each haploid genomic equivalent weighs 3.3 pg.^49^ As reported in the previous article,^23^ the ΔctDNA was calculated by dividing the ctDNA concentration at on-treatment timepoint with the baseline ctDNA concentration in our cohort. If ctDNA was not detectable at baseline, the limit of detection for this cohort was used as the ctDNA concentration. Considering the intra-day ctDNA variation, the function of “surv_cutpoint” in the survminer R package was used to determine the optimal cutoff value to define the threshold for ctDNA molecular response. The cut-point ≤0.8-fold decrease from baseline was considered as ctDNA molecular response. Also, ΔVAF was calculated as the VAF at on-treatment timepoint divided by the baseline VAF.

### Statistical analysis

For PFS analysis, the log-rank test was used to compare Kaplan–Meier survival curves, and Cox proportional hazards regression models were used to generate hazard ratios. Univariate and multivariate Cox proportional hazards regression analyses were performed to identify independent factors corrected with prognosis.

The multimodal model predicting DCB after ICI treatment was established by using binary logistic regression, with our data as the training set and the DIREct-On data as the validation set.^23^ The optimal ROC corner point approach (Youden’s J) was used to select the optimal parameter and cut-points for the multimodal model.^50^ These cut-points were then applied to the validation set. Moreover, a fivefold cross-validation process was repeated 20 times to measure the accuracy of individual parameters. Two-tailed Mann–Whitney tests were used to compare distributions, and two-sided Fisher’s exact tests were used to compare proportions. Bonferroni correction was performed for multiple testing. A two-sided *P* value < 0.05 was considered statistically significant. Statistical analyses were performed using R (v4.3.2) and GraphPad Prism (v8.0.1) software.

## Supplementary information

Supplementary_Figures _Tables

TRACELib002 Study Protocol

## Data Availability

The data generated in this study were deposited in the National Genomics Data Central (NGDC) database under accession number HRA007985 and data were available upon reasonable request to the corresponding author.
