# Supplementary material for: Noninvasive early identification of durable clinical benefit from immune checkpoint inhibition: a prospective multicenter study (NCT04566432)
Source: Signal Transduct Target Ther. 2024 Dec 16;9:350. doi: 10.1038/s41392-024-02060-3 (PMC11646999; doi:10.1038/s41392-024-02060-3)
Supplement: Supplementary file 1 — Supplementary_Figures _Tables [file 41392_2024_2060_MOESM1_ESM.docx]

Supplementary Materials for

Noninvasive early identification of durable clinical benefit from immune checkpoint inhibition: a prospective multicenter study (NCT04566432)

Xinghao Ai; Bo Jia; Zhiyi He; Junping Zhang; Minglei Zhuo ; Jun Zhao; Zhe Wang; Jiexia Zhang; Zaiwen Fan; Xiaotong Zhang; Chong Li; Feng Jin; Ziming Li; Xia Ma; Hao Tang; Xiang Yan; Wei Li; Yuanyuan Xiong; Huan Yin; Rongrong Chen; Shun Lu

Correspondence to: [shunlu@sjtu.edu.cn](mailto:shunlu@sjtu.edu.cn), chenrr@geneplus.org.cn

This PDF file includes:

Supplementary Figures. 1 to 6

Supplementary Tables 1


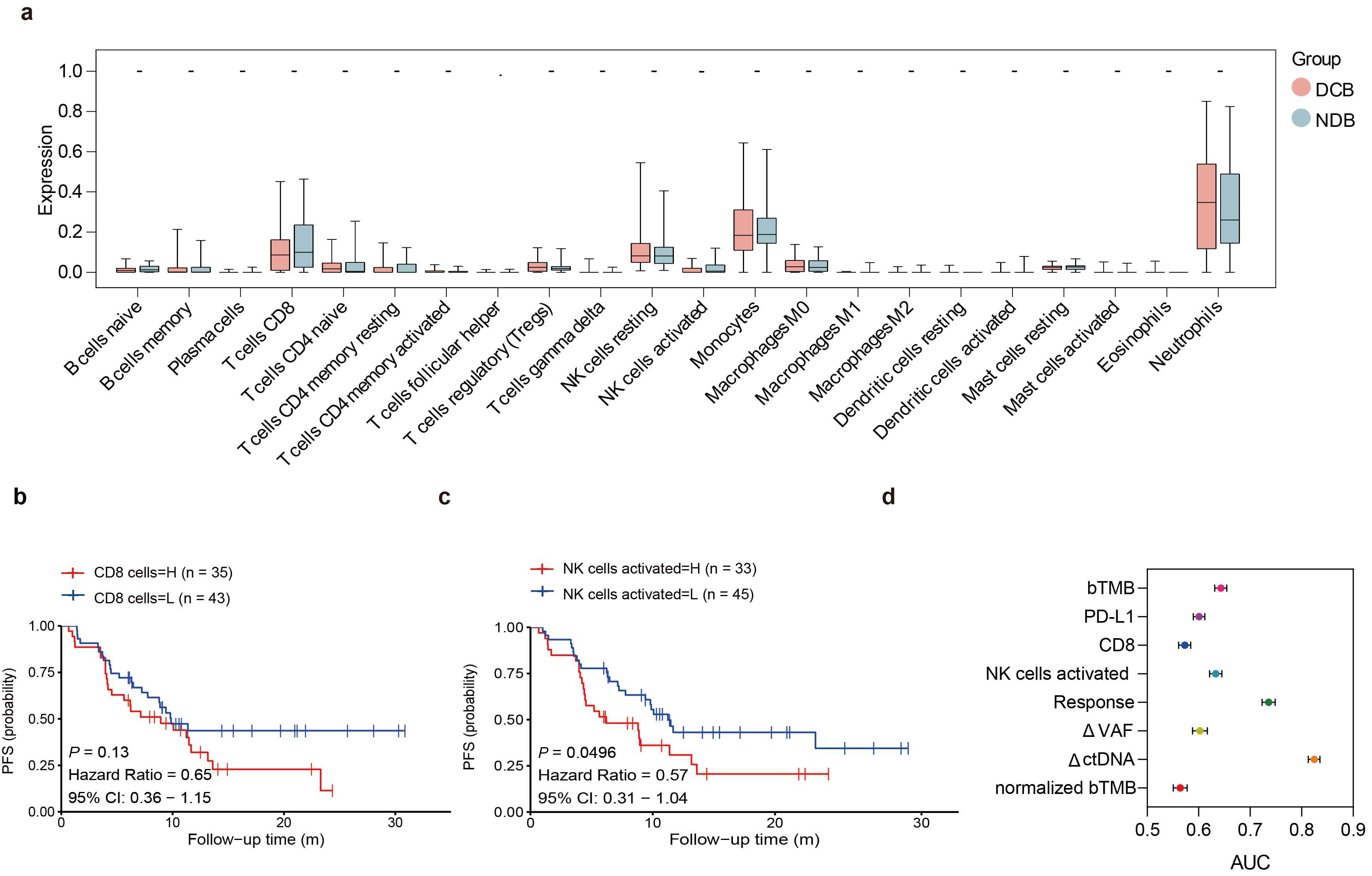


Supplementary Figure. 1. Pre-treatment activated NK cells predicts response to ICI.

**a** Pre-treatment relative multiple cell fraction in circulation of DCB and NDB with available CIBERSORTx immune profiling. There was no difference in cell fraction between DCB and NDB groups. **b** PFS analysis based on the CD8 cells level. There was no difference in PFS between high and low level of CD8 cells. **c** PFS analysis based on the activated NK cells level. There was only a limited difference in PFS between high and low level of activated NK cells. **d** AUC for individual parameters generated by 5 fold cross-validation ROC analysis. PFS: progression-free survival; bTMB: blood-based TMB; DCB: durable clinical benefit; NDB: no durable benefit; AUC: area under the receiver operating characteristic curve; *P* < 0.05 represents statistical significance.


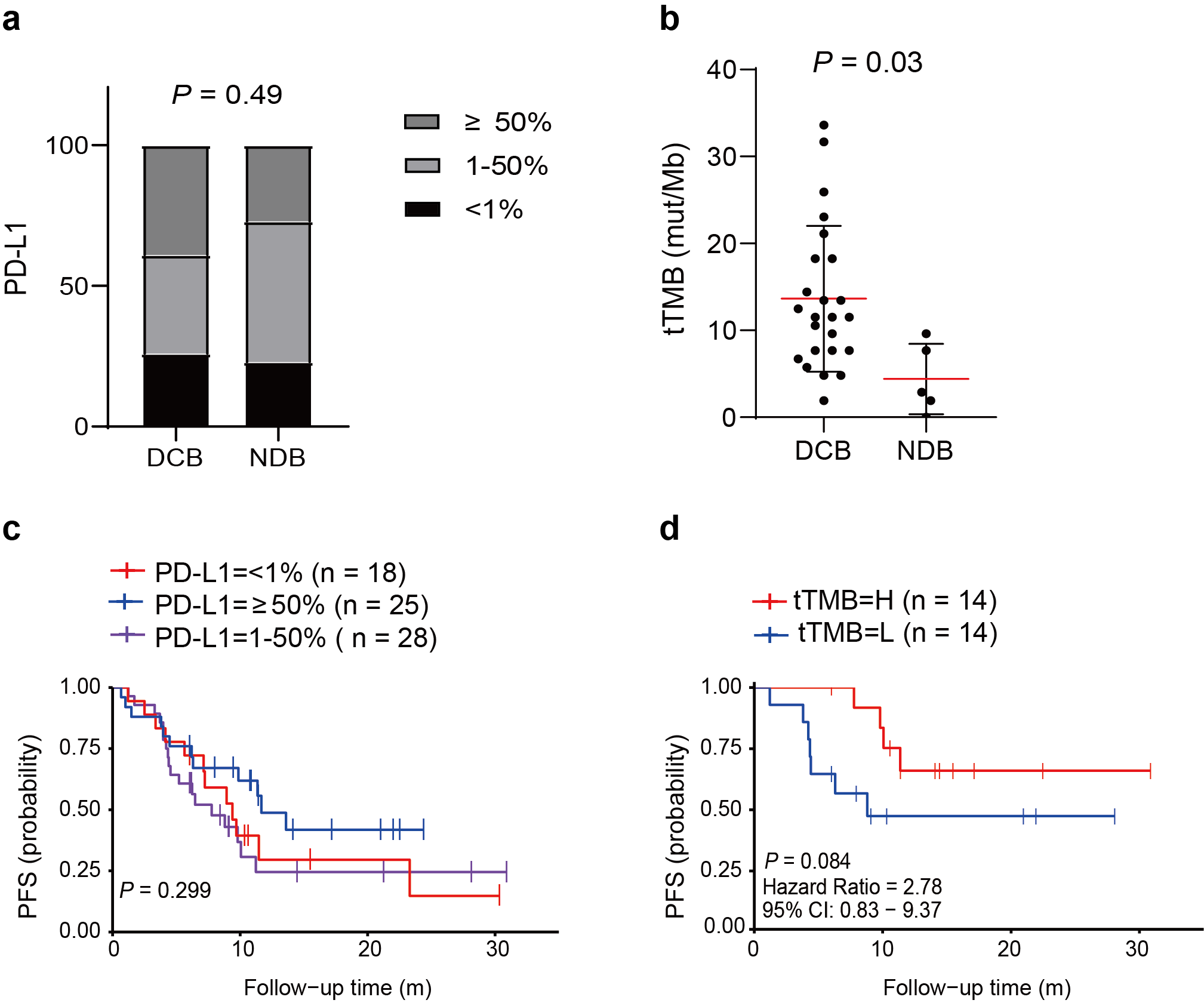


Supplementary Figure. 2. Pre-treatment tissue characteristics can not predict response to ICI.

**a** There was no difference in PD-1 expression between DCB and NDB groups. **b** The levels of tTMB were higher in DCB compared to the NDB group. **c-d**  PFS analysis based on PD-L1 expression and tTMB level, both of them had no significant correlation with PFS. PFS: progression-free survival; tTMB: tumor mutational burden; DCB: durable clinical benefit; NDB: no durable benefit. *P* < 0.05 represents statistical significance.


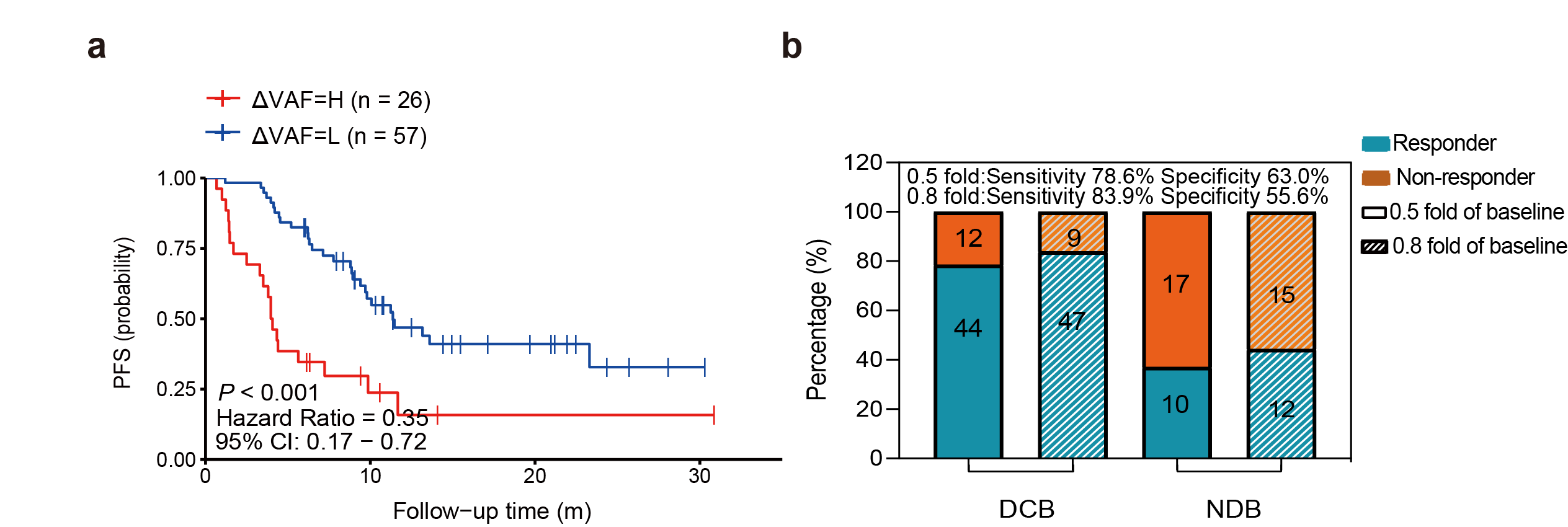


Supplementary Figure. 3. The change of VAF predicts response to ICI.

**a** PFS analysis based on the ΔVAF level at baseline. Patients with low ΔVAF had longer PFS compared with patients who have high ΔVAF. **b** Outcomes of 83 patients stratified by ΔVAF. And the ΔVAF had good sensitivity and specificity to identify patients with DCB. PFS: progression-free survival; DCB: durable clinical benefit; NDB: no durable benefit. *P* < 0.05 represents statistical significance.


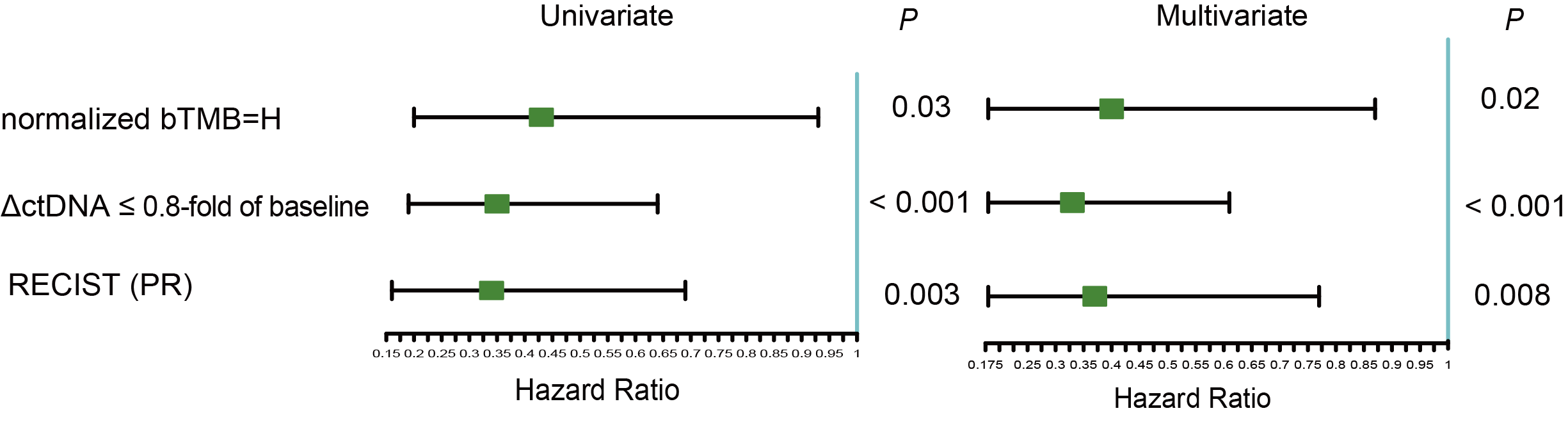


Supplementary Figure. 4. Correlation analysis between ΔctDNA, normalized bTMB and RECTSI response.

Univariate and multivariate analyses based on Cox regression analysis showed ΔctDNA, normalized bTMB and RECTSI response were independent risk factors for prognosis. bTMB: blood-based TMB; RECIST: The response evaluation criteria in solid tumors. *P* < 0.05 represents statistical significance.


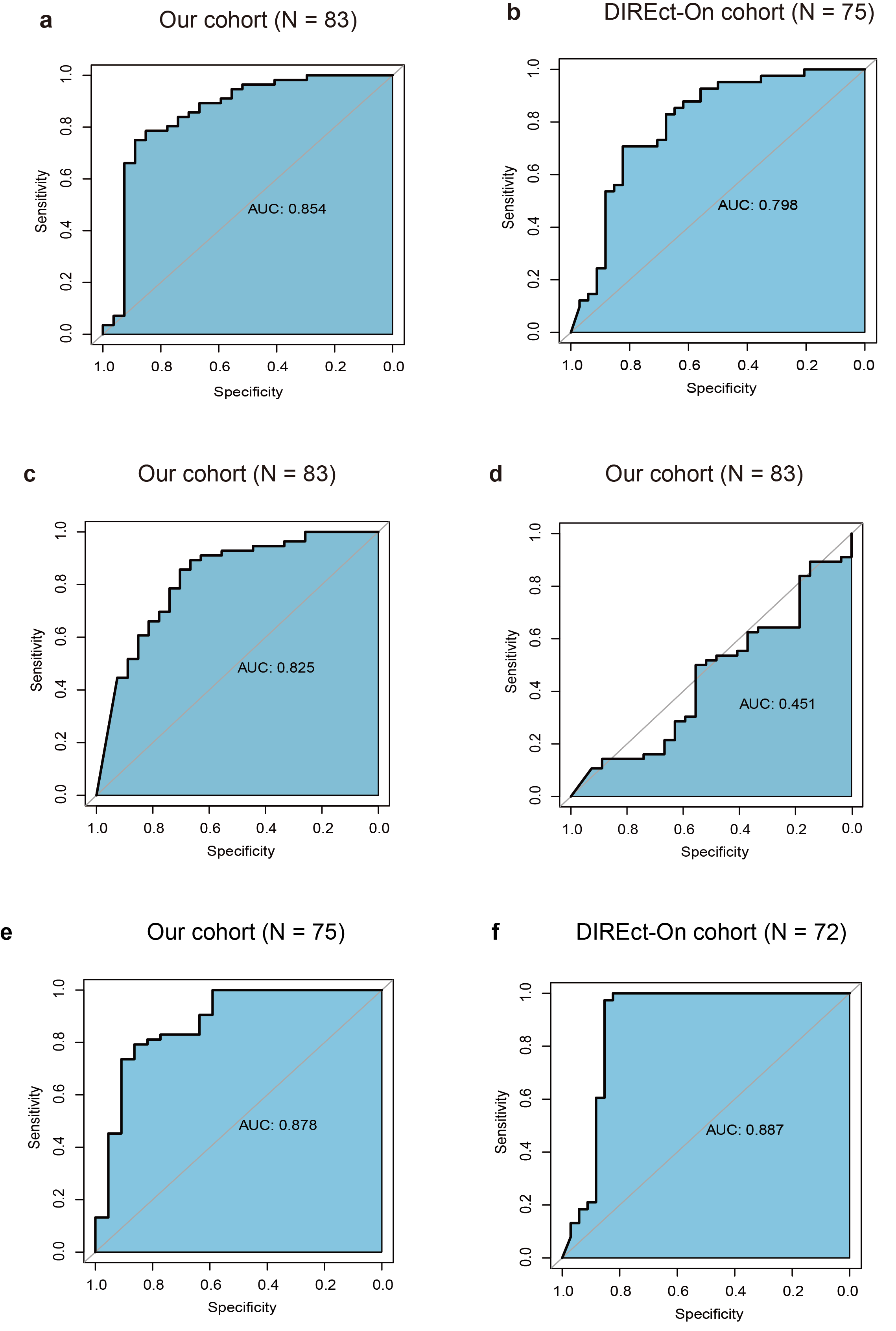


Supplementary Figure. 5. Multimodal model enables fully noninvasive outcome classification.

**a** Two parameters (normalized bTMB and ΔctDNA) predict DCB with AUC of 0.854 in our discovery cohort. **b** Two parameters (normalized bTMB and ΔctDNA) predict DCB with AUC of 0.798 in DIREct-On validation cohort. **c** ΔctDNA predict DCB with AUC of 0.825 in our discovery cohort. **d** Normalized bTMB predict DCB with AUC of 0.451 in our discovery cohort. **e** Three parameters (normalized bTMB, ΔctDNA and the first RECIST response) predict DCB with AUC of 0.878 in our discovery cohort. **f** Three parameters (normalized bTMB, ΔctDNA and the first RECIST response) predict DCB with AUC of 0.887 in DIREct-On validation cohort. AUC: area under the receiver operating characteristic curve.


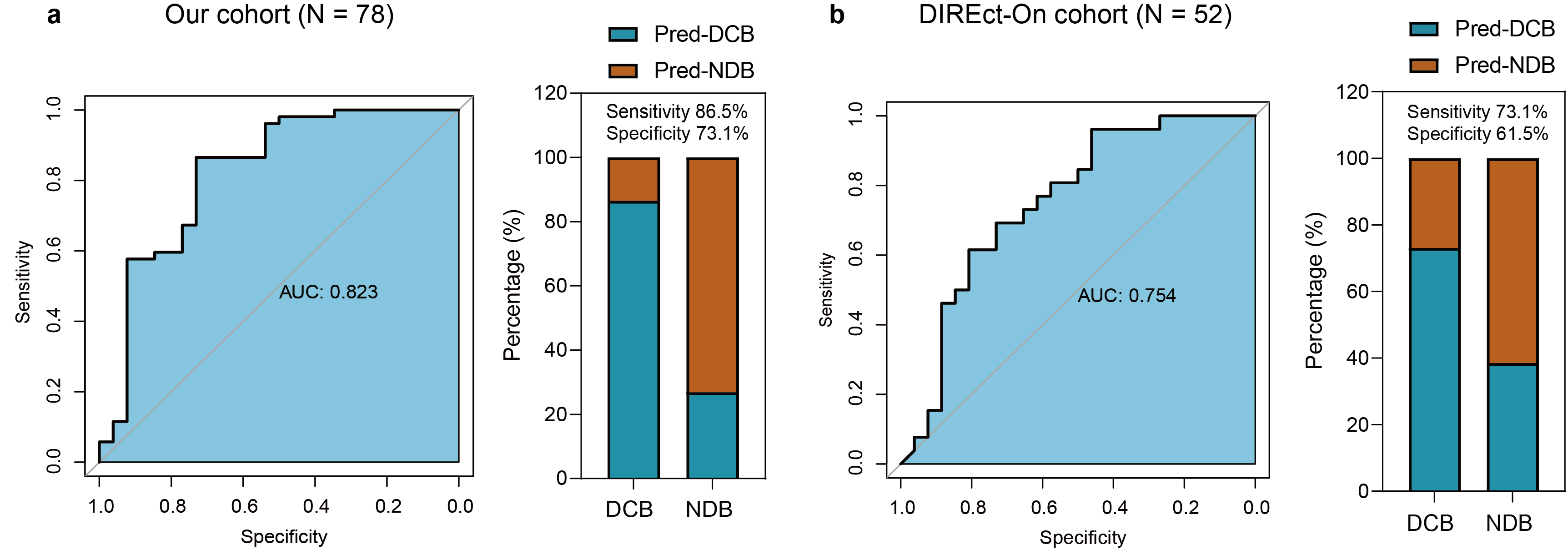


Supplementary Figure. 6. Activated NK cells impaired the predictive ability of the model.

**a** Three parameters (NK cells, ΔctDNA and the first RECIST response) were used to predict DCB with AUC of 0.823, a sensitivity of 86.5% and a specificity of 73.1% in our discovery cohort. Stacked column chart showing the proportion of patients predicted to achieve DCB (Pred-DCB) or NDB (Pred-NDB) by the model. **b** Three parameters (NK cells, ΔctDNA and the first RECIST response) were used to predict DCB with AUC of 0.754, a sensitivity of 73.1% and a specificity of 61.5% in DIREct-On validation cohort. Stacked column chart showing the proportion of patients with pred-DCB or Pred-NDB by the model. DCB: durable clinical benefit; NDB: no durable benefit.

Supplementary Table 1. Variables comprising the final predictive model for progression-free survival.

|  | **Hazard ratio (95% CI)** | **Importance*** |
| --- | --- | --- |
| ΔctDNA (>0.8 fold form baseline) | 1(ref) | 24 |
| ΔctDNA (≤0.8 fold form baseline) | 0.33 (0.18-0.61) |  |
| Normalized bTMB (L) | 1 (ref) | 12 |
| Normalized bTMB (H) | 0.42 (0.19-0.92) |  |
| Response (SD/PD) | 1 (ref) | 18 |
| Response (PR) | 0.34 (0.16-0.74) |  |

*Defined by the χ2 Wald statistic.
