# Supplementary material for: Noninvasive early identification of durable clinical benefit from immune checkpoint inhibition: a prospective multicenter study (NCT04566432)
Source: Signal Transduct Target Ther. 2024 Dec 16;9:350. doi: 10.1038/s41392-024-02060-3 (PMC11646999; doi:10.1038/s41392-024-02060-3)
Supplement: Supplementary file 2 — TRACELib002 Study Protocol [file 41392_2024_2060_MOESM2_ESM.pdf]

Study Protocol

Therapeutic ResistAnce and Clonal Evolution Assessed With Liquid Biopsy in ICIs Treated NSCLC Patients

Trial Number: TRACELib002

Registration Number: NCT04566432

IRB Approval Number: KS2017

Sponsor: Geneplus-Beijing Co. Ltd.

Principal investigator: Shun Lu

Version: 2.0

Contents

1 Study background and rationale....3
1.1 Study background....3
1.2 Study rationale....4
1.3 Exploratory study....4
2 Study objectives and study endpoints....4
3 Study design....4
3.1 description of study design....4
3.2 Definition of end of study....5
3.3 Duration and sample size....5
3.4 Ethical application....6
4 Study population....6
4.1 Inclusion criteria....6
4.2 Exclusion criteria....6
5 Study procedure and statistical analysis plan....6
5.1 Study treatment....6
5.2 Study flow chart....7
5.3 Test schedule....7
5.4 Patient efficacy assessment....8
5.5 Statistical analysis plan....8
6 Data collection and management....9
7 Ethical considerations and management procedures....10
7.1 Regulatory and ethical compliance....10
7.2 Responsibilities of the investigator and IRB/IEC/REB....10
7.3 Informed consent procedures....11
7.4 Publication of study protocol and results....11
7.5 Articles published by the investigator....11
7.6 Confidentiality of study documents and patient records....12
8 Protocol amendment....12
References....13

1 Study background and rationale

1.1 Study background

Lung cancer is one of the most prevalent malignant tumors and the leading cause of cancer deaths in China. According to data published by the National Cancer Center in 2015, the five-year prevalence of lung cancer in China from 2006 to 2011 was 130.2 per 100,000 individuals. Approximately 80-85% of lung cancers are non-small cell lung cancer (NSCLC), with lung adenocarcinoma accounting for about 55% of cases. Most NSCLCs do not present with obvious symptoms in the early stages, leading to late-stage diagnosis in approximately 70% of patients, at which point the five-year survival rate is only 15% [1].

With the approval of an increasing number of targeted therapies and the wide use of next-generation sequencing (NGS) and other genetic testing technologies in the clinic, patients with lung cancer harboring targetable mutations such as *EGFR*, *ALK*, *ROS1*, and *MET* have got significant better treatment outcomes. However, for the NSCLC patients without identifiable driver gene mutations, chemotherapy remains the major treatment choice.

Clonal architecture is fundamental for the understanding of cancer biology and therapy. In the previous TRACELib001 (NCT03059641) study, we identified that circulating tumor DNA (ctDNA) could describe the clonal architecture of advanced NSCLC, and *EGFR* mutations do not always make up a dominant clone in advanced NSCLC. Moreover, EGFR clonal architecture was an independent prognostic indicator of the efficacy of EGFR-tyrosine kinase inhibitors[1]. Whether this finding could be extended to other targeted therapy or even immunotherapy was unknown.

In recent years, PD-1 inhibitors such as nivolumab, pembrolizumab, and camrelizumab, as well as PD-L1 inhibitors such as atezolizumab and durvalumab, have demonstrated survival benefits compared to chemotherapy in various studies including Checkmate, Keynote, and Impower [2-8]. Both the FDA and the National Medical Products Administration (NMPA) have approved multiple immune checkpoint inhibitors (ICIs) for clinical use [9-11], however, only a subset of patients experience long-term benefits from these treatments [12,13]. Moreover, currently imaging assessments, tumor markers,

tumor mutation burden (TMB) [14-16], PD-L1 expression[17,18], and circulating tumor DNA (ctDNA) [19] are insufficient to predict therapeutic efficacy from PD-1/PD-L1 inhibitors[20].

1.2 Study rationale

Recently, a research team from Stanford University established the DIREct-On model[21], which combines blood TMB (bTMB), changes in ctDNA, and peripheral blood CD8+ T cells to predict whether patients will benefit from long-term treatment with PD-1/PD-L1 inhibitors. In the retrospective study, DIREct-On model could predict patients who would achieve durable clinical benefit (DCB, over 6 months) with high accuracy[21]. Inspired by this study, we designed this prospective multicenter clinical trial to explore multimodal models that were more feasible in the clinical routine for predicting DCB of ICI treatments in non-EGFR/ALK NSCLC patients.

1.3 Exploratory study

While screening *EGFR*, *ALK* mutations, we might accidently identify patients with actionable *ROS1* fusion, or *MET* exon 14 skipping mutation as well as *ALK* fusion, thus we also explore the clonal evolution with liquid biopsy in these patients treated with corresponding TKI.

2 Study objectives and study endpoints

Study objectives	Study endpoints
**Primary study objective**<br>To establish a multimodal model to predict the efficacy of ICI treatments	Primary study endpoint<br>PFS
**Secondary study objective**<br>To explore mutations associated with hyperprogression and severe toxic side effects in ICI Therapy	Secondary study endpoint<br>OS
**Exploratory study objective**<br>To investigate clonal evolution of tumor cells treated with ICI or TKI	

3 Study design

3.1 description of study design

This study is a prospective multi-center real-world study designed to explore

multimodal models to predict the efficacy of first-line ICI treatments, as well as the clonal evolution of tumor cells treated with ICI or TKI in patients with advanced Non-Small-Cell Lung Cancer (Stage IIIB-IV, 8th edition TNM staging). ctDNA and PBMC-RNA analysis will be performed at preset time for patients treated with first-line ICI treatment. For the exploratory study subgroup – patients with *ROS1* fusion, or *MET* exon 14 skipping mutation as well as *ALK* fusion and treated with corresponding TKI, only ctDNA analysis will be performed at preset time.

3.2 Definition of end of study

The end of the study is defined as over 70% of the patients had a progressive disease (PD) in their targeted lesion of the study. There would be interim data analysis and final study data analysis.

3.3 Duration and sample size

The original plan was to commence in July 2020 and conclude participant enrollment by June 2023. (However, due to the COVID-19 pandemic, the enrollment is still ongoing)

The planned enrollment for this study was 250 participants from multiple centers, including *EGFR/ALK* negative NSCLC patients who received first-line ICI treatment, and NSCLC patients with *ROS1* fusion, *ALK* fusion or *MET* exon 14 skipping mutation treated with corresponding TKI. The sample size estimation was listed as the following: For the ICI subgroup, a novel clinical prediction model based on binary logistic regression was to be developed. Referring to the DIREct-On study[21], which enrolled 72 patients and developed an excellent model with 3 variants (peripheral CD8 T cell levels, ctDNA dynamics and ctDNA adjusted TMB) as input, we also planned 3 variants for the modeling. The prevalence of durable clinical benefit (DCB) from ICI treatments was around 30%-70%[16,21]. With the criterion of 10 Events Per Variable (10 EPV), the minimal sample size required for the study was 60-100 patients.

For the targeted therapy subgroup, the sample size estimation was basically according to the prevalence of each target, as it was a descriptive study of clonal evolution. We planned to enrolled 100 patients treated with ALK-TKI, 25 patients treated with MET-TKI and 25 patients treated with ROS1-TKI.

3.4 Ethical application

The study protocol was submitted to and approved by the Hospital Ethics Committee. All patients are required to sign an informed consent form before enrollment.

4 Study population

4.1 Inclusion criteria

1. Provision of informed consent
2. Newly diagnosed and histological or cytological confirmed stage IIIB-IV lung adenocarcinoma or squamous cell carcinoma patients according to the AJCC staging system. The stage IV lung cancer and brain metastasis can be diagnosed by imaging and enhanced CT respectively
3. No EGFR mutation in tissue and ctDNA
4. Received immune checkpoint inhibitors as the first line therapy
5. ECOG performance status 0-2 with expected more than 6 months of survival time
6. Willingness to comply with required protocols and give permission to use the data for clinical research and products development

4.2 Exclusion criteria

1. Patients have other primary cancers
2. Patients have symptomatic brain metastasis, complications that are associated with brain metastasis or cognitive disorders
3. Patients failed in either plasma or tissue sample QC

5 Study procedure and statistical analysis plan

5.1 Study treatment

Patients were treated according to clinical routine. *EGFR/ALK* negative NSCLC patients who received first-line ICI treatment, and NSCLC patients with *ROS1* fusion, *ALK* fusion or *MET* exon 14 skipping mutation treated with corresponding TKI were eligible for enrollment in the study.

5.2 Study flow chart

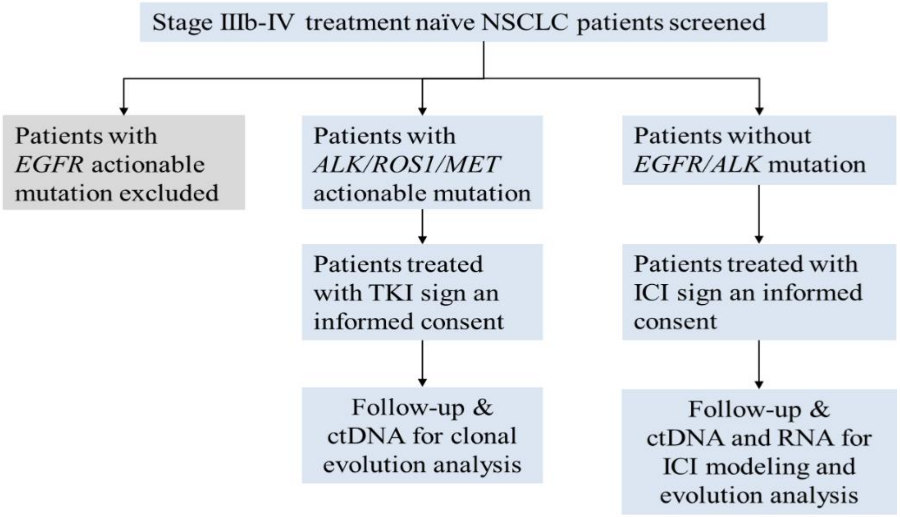

Figure 1 Study flow

5.3 Test schedule

Table 1 Test schedule

Entry	Screening 1		Screening 2	ICI/TKI treatment	After treatment ends/quit		After-PD follow-up
	Within 3 weeks	Within 1 week	Tumor mutation testing	After 1-2 cycles	Treatment ends/quit	~30 days after ends or quit	Per 3 months
Clinic visit	×		×	×	×	×	
Informed consent	×						
Demographic information	×						
Medical history	×						
Physical examination		×	×	×	×		×
Laboratory examination		×	×	×	×	×	×
Radiological assessment	×			Every 2-4 cycles	×		×
ECG/Echocardiogram		×		×	×		
ECOG PS		×		×	×	×	×
Inclusion/exclusion		×	×				
Tumor/blood samples		×		×	×		
Side effects			×	×	×		
Response assessment	×		×	×	×	×	×
OS follow-up			×	×	×	×	×

5.4 Patient efficacy assessment

Efficacy assessments included PFS, OS. Primary efficacy indicators:

5.4.1 Primary endpoint

Progress Free survival (PFS): PFS for stage IIIB-IV NSCLC who received first-line systemic therapy was defined from the start of first-line systemic therapy to disease progression or death. Disease progression was defined according to RECIST 1.1 criteria. The date on that patients receive their last imaging evaluation while alive and progress-free is used as the cutoff.

5.4.2 Secondary endpoints

Overall survival (OS): OS for first-line treatment, from the start of first-line treatment until death, and for surviving patients, until the date of last follow-up.

5.5 Statistical analysis plan

5.5.1 Sample size calculation

For the ICI subgroup, a novel clinical prediction model based on binary logistic regression was to be developed. Referring to the DIREct-On study[21], which enrolled 72 patients and developed an excellent model with 3 variants (peripheral CD8 T cell levels, ctDNA dynamics and ctDNA adjusted TMB) as input, we also planned 3 variants for the modeling. The prevalence of durable clinical benefit (DCB) from ICI treatments was around 30%-70%[16,21]. With the criterion of 10 Events Per Variable (10 EPV), the minimal sample size required for the study was 60-100 patients.

For the targeted therapy subgroup, the sample size estimation was basically according to the prevalence of each target, as it was a descriptive study of clonal evolution. We planned to enrolled 100 patients treated with ALK-TKI, 25 patients treated with MET-TKI and 25 patients treated with ROS1-TKI.

5.5.2 Grouping method

For the ICI subgroup, patients will be divided into 2 groups with 6 months PFS as the cutoff, with PFS of durable clinical benefit (DCB) group ≥ 6 months and non-durable benefit (NDB) <6 months, referring to the DIREct-On study[21].

For the targeted therapy subgroup, further grouping includes: primary resistance group (progressed in 3 months), long term benefits (PFS ≥ 36 months), and the rest which

could be further divided into PFS ≥ medium reported PFS or not.

5.5.3 Statistical analysis

The database will be transferred to the statistical analysts for statistical analysis in accordance with the plan requirements. The statistical unit prepared a statistical analysis report to the principal investigator who are obliged to complete the study results.

5.5.3.1 Baseline and demographic characteristics

Baseline demographic characteristics, baseline clinical characteristics, and mutation profiling will be descriptively analyzed for all enrolled patients. For continuous measures, mean, standard deviation, range and median will be calculated; absolute values, frequencies and percentages were calculated.

5.5.3.2 Primary endpoint analysis

PFS: The Kaplan-Meier method will be used to describe the 25%, 75%, and median event occurrence times, along with a 95% two-sided confidence interval, and to plot the Kaplan-Meier survival curve up to the time of data cutoff. The COX proportional risk regression model was used for each covariate to test the influence factors related to the prognosis of recurrence in terms of hazard ratios (HR) and their estimated 95% two-sided confidence intervals.

For the ICI subgroup, recapitulation of the DIREct-On model[21] will be tried and binary logistic regression approached will be used to develop novel multimodal models.

5.5.3.3 Secondary endpoint analysis

OS: The Kaplan-Meier method will be used to describe the 25%, 75%, and median event occurrence times, along with a 95% two-sided confidence interval, and to plot the Kaplan-Meier survival curve up to the time of data cutoff. The COX proportional risk regression model will be used for each covariate to test the influence factors related to the prognosis of recurrence in terms of hazard ratios (HR) and their estimated 95% two-sided confidence intervals.

6 Data collection and management

The completion and transfer of Case Report Form (CRFs) are based on the most original records for research medical records, ensuring proper preservation. The case report

form is taken from the study medical record and is completed by the investigators, who must complete the case report form for each enrolled case. The completed case report form is reviewed by the clinical supervisor and the first link is submitted to the data statistics unit for data entry and management. The content of the case report form will not be modified after the transfer of the first link to database.

Specialized personnel will be appointed to manage the data. The data manager will complete the Data Rating Questionnaire (DRQ) and send a query to the investigator through the Clinical Supervisor, the investigator is warrant to response the query as soon as possible, and the data manager will correct the data based on the investigator's response, and may resend the DRQ again if necessary.

The data manager performs data verification of the data and issues a data verification report, which is reviewed by the principal investigator, the sponsor, the statistical analyst and the data manager, and the data set for statistical analysis is determined before the data is locked.

Ensure that research data is true, accurate and reliable. Strictly control the whole process of data collection, processing, to statistical analysis and reporting. In accordance with the ALCOA+ data quality evaluation principles, ensure data traceability, completeness, consistency, and accuracy.

7 Ethical considerations and management procedures

7.1 Regulatory and ethical compliance

The clinical study design, implementation and reporting shall be carried out in accordance with the three-party coordinated guidelines of The International Council for Harmonization (ICH) of Technical Requirements for Pharmaceuticals for Human Use, relevant applicable local regulations (including EU Directive 2001/20/EC and 21 CFR) and ethical principles formulated in accordance with the "Declaration of Helsinki".

7.2 Responsibilities of the investigator and IRB/IEC/REB

Before the start of the study, the study protocol and the proposed ICF must be reviewed and approved by the Institutional Review Board (IRB)/Independent Ethics Committee (IEC)/Research Ethics Board (REB) composed of relevant qualified personnel. Before

the start of the study, the investigator is required to sign the signature page of the protocol to confirm that he/she agrees to carry out the study in accordance with these documents and all the instructions and procedures required by the study protocol, and to provide the relevant data and records to IRB/IEC/REB.

7.3 Informed consent procedures

Patients may be enrolled into the study only after eligible patients have provided written (in accordance with the law or regulations with witnesses present) ICF approved by IRB/IEC/REB.

Any study-specified procedures (i.e., all procedures specified in the study protocol) can only be performed after informed consent is obtained. The process of obtaining the informed consent of the patient should be recorded in the patient's source document. The date on which the subject ICF is actually obtained will be recorded in the patient's CRF.

This study will use a separate document to provide the investigator with a proposed ICF, which will be implemented in accordance with the guidelines and regulations in the international unified standards for clinical trial management, and the ICF is considered to be applicable to this study. If the investigator proposes a revision to the ICF, the IRB/IEC/REB's approval must be obtained before using the revised ICF, and a copy of the approved version must be submitted to the sponsor and the monitor of each company.

7.4 Publication of study protocol and results

The sponsor promises to report the study results in accordance with high ethical standards of publication, including timely publishing clinical trial results, regardless of the outcome. The sponsor shall ensure that the key design content of this study protocol is announced in a database open to the public, such as www.clinicaltrials.gov, before the start of the study.

7.5 Articles published by the investigator

In order to prevent inadvertent disclosure of confidential information and protect intellectual property, the investigator must notify the main responsible persons of each company before submission for publication or disclosure in other forms, so that each company has the opportunity to review and discuss the articles prepared for publication

or other materials prepared for disclosure.

The sponsor supports the overall publication of the multicenter trial and does not support the separate publication of single-center data. The authorship will be decided by both parties or by tripartite agreement.

7.6 Confidentiality of study documents and patient records

The study staff must ensure that the patient participates in the study anonymously, and the patient must not be identified by name in the documents submitted to each company. The signed ICF and patient enrollment records must be strictly confidential and can only be identified for patients at each study site.

8 Protocol amendment

Any protocol amendment should be handed over to the sponsor for amendment, and a written protocol amendment shall be generated and submitted to the IRB/IEC/REB for approval. The revised protocol can only be implemented prior to IRB/IEC/REB approval if it is for ensuring the patient's safety. In this case, the investigator should inform the sponsor of the measures taken and report it to the IRB/IEC/REB of the study site.

References

1 Ai, X. *et al.* Clonal Architecture of EGFR Mutation Predicts the Efficacy of EGFR-Tyrosine Kinase Inhibitors in Advanced NSCLC: A Prospective Multicenter Study (NCT03059641). *Clin Cancer Res* **27**, 704-712, doi:10.1158/1078-0432.CCR-20-3063 (2021).

2 Brahmer, J. *et al.* Nivolumab versus Docetaxel in Advanced Squamous-Cell Non-Small-Cell Lung Cancer. *The New England journal of medicine* **373**, 123-135, doi:10.1056/NEJMoa1504627 (2015).

3 Borghaei, H. *et al.* Nivolumab versus Docetaxel in Advanced Nonsquamous Non-Small-Cell Lung Cancer. *The New England journal of medicine* **373**, 1627-1639, doi:10.1056/NEJMoa1507643 (2015).

4 Vokes, E. E. *et al.* Nivolumab versus docetaxel in previously treated advanced non-small-cell lung cancer (CheckMate 017 and CheckMate 057): 3-year update and outcomes in patients with liver metastases. *Annals of oncology : official journal of the European Society for Medical Oncology* **29**, 959-965, doi:10.1093/annonc/mdy041 (2018).

5 Horn, L. *et al.* Nivolumab Versus Docetaxel in Previously Treated Patients With Advanced Non-Small-Cell Lung Cancer: Two-Year Outcomes From Two Randomized, Open-Label, Phase III Trials (CheckMate 017 and CheckMate 057). *Journal of clinical oncology : official journal of the American Society of Clinical Oncology* **35**, 3924-3933, doi:10.1200/JCO.2017.74.3062 (2017).

6 Herbst, R. S. *et al.* Pembrolizumab versus docetaxel for previously treated, PD-L1-positive, advanced non-small-cell lung cancer (KEYNOTE-010): a randomised controlled trial. *Lancet* **387**, 1540-1550, doi:10.1016/S0140-6736(15)01281-7 (2016).

7 Fehrenbacher, L. *et al.* Updated Efficacy Analysis Including Secondary Population Results for OAK: A Randomized Phase III Study of Atezolizumab versus Docetaxel in Patients with Previously Treated Advanced Non-Small Cell Lung Cancer. *Journal of thoracic oncology : official publication of the International Association for the Study of Lung Cancer* **13**, 1156-1170, doi:10.1016/j.jtho.2018.04.039 (2018).

8 Rittmeyer, A. *et al.* Atezolizumab versus docetaxel in patients with previously treated non-small-cell lung cancer (OAK): a phase 3, open-label, multicentre randomised controlled trial. *Lancet* **389**, 255-265, doi:10.1016/S0140-6736(16)32517-X (2017).

9 Pai-Scherf, L. *et al.* FDA Approval Summary: Pembrolizumab for Treatment of Metastatic Non-Small Cell Lung Cancer: First-Line Therapy and Beyond. *The oncologist* **22**, 1392-1399, doi:10.1634/theoncologist.2017-0078 (2017).

10 Sul, J. *et al.* FDA Approval Summary: Pembrolizumab for the Treatment of Patients With Metastatic Non-Small Cell Lung Cancer Whose Tumors Express Programmed Death-Ligand 1. *The oncologist* **21**, 643-650, doi:10.1634/theoncologist.2015-0498 (2016).

11 Weinstock, C. *et al.* U.S. Food and Drug Administration Approval Summary: Atezolizumab for Metastatic Non-Small Cell Lung Cancer. *Clinical cancer research : an official journal of the American Association for Cancer Research* **23**, 4534-4539, doi:10.1158/1078-0432.CCR-17-0540 (2017).

12 Carbone, D. P. *et al.* First-Line Nivolumab in Stage IV or Recurrent Non-Small-Cell Lung Cancer. *The New England journal of medicine* **376**, 2415-2426, doi:10.1056/NEJMoa1613493 (2017).

13 Langer, C. J. *et al.* Carboplatin and pemetrexed with or without pembrolizumab for

advanced, non-squamous non-small-cell lung cancer: a randomised, phase 2 cohort of the open-label KEYNOTE-021 study. *The Lancet. Oncology* **17**, 1497-1508, doi:10.1016/S1470-2045(16)30498-3 (2016).

14 Rizvi, N. A. *et al.* Cancer immunology. Mutational landscape determines sensitivity to PD-1 blockade in non-small cell lung cancer. *Science* **348**, 124-128, doi:10.1126/science.aaa1348 (2015).

15 Yarchoan, M., Hopkins, A. & Jaffee, E. M. Tumor Mutational Burden and Response Rate to PD-1 Inhibition. *The New England journal of medicine* **377**, 2500-2501, doi:10.1056/NEJMc1713444 (2017).

16 Rizvi, H. *et al.* Molecular Determinants of Response to Anti-Programmed Cell Death (PD)-1 and Anti-Programmed Death-Ligand 1 (PD-L1) Blockade in Patients With Non-Small-Cell Lung Cancer Profiled With Targeted Next-Generation Sequencing. *J Clin Oncol* **36**, 633-641, doi:10.1200/JCO.2017.75.3384 (2018).

17 Reck, M. *et al.* Pembrolizumab versus Chemotherapy for PD-L1-Positive Non-Small-Cell Lung Cancer. *The New England journal of medicine* **375**, 1823-1833, doi:10.1056/NEJMoa1606774 (2016).

18 Mok, T. S. K. *et al.* Pembrolizumab versus chemotherapy for previously untreated, PD-L1-expressing, locally advanced or metastatic non-small-cell lung cancer (KEYNOTE-042): a randomised, open-label, controlled, phase 3 trial. *Lancet* **393**, 1819-1830, doi:10.1016/S0140-6736(18)32409-7 (2019).

19 Anagnostou, V. *et al.* Dynamics of Tumor and Immune Responses during Immune Checkpoint Blockade in Non-Small Cell Lung Cancer. *Cancer research* **79**, 1214-1225, doi:10.1158/0008-5472.CAN-18-1127 (2019).

20 Camidge, D. R., Doebele, R. C. & Kerr, K. M. Comparing and contrasting predictive biomarkers for immunotherapy and targeted therapy of NSCLC. *Nature reviews. Clinical oncology* **16**, 341-355, doi:10.1038/s41571-019-0173-9 (2019).

21 Nabet, B. Y. *et al.* Noninvasive Early Identification of Therapeutic Benefit from Immune Checkpoint Inhibition. *Cell* **183**, 363-376 e313, doi:10.1016/j.cell.2020.09.001 (2020).
